# Charting a Path to the Quintuple Aim: Harnessing AI to Address Social Determinants of Health

**DOI:** 10.3390/ijerph21060718

**Published:** 2024-05-31

**Authors:** Yash B. Shah, Zachary N. Goldberg, Erika D. Harness, David B. Nash

**Affiliations:** 1 Sidney Kimmel Medical College, Thomas Jefferson University, Philadelphia, PA 19107, USAgoldberg.zach11@gmail.com (Z.N.G.);; 2 Jefferson College of Population Health, Philadelphia, PA 19107, USA

**Keywords:** social determinants of health, health-related social needs, artificial intelligence, ChatGPT, disparities in care, healthcare management, quintuple aim

## Abstract

The Quintuple Aim seeks to improve healthcare by addressing social determinants of health (SDOHs), which are responsible for 70–80% of medical outcomes. SDOH-related concerns have traditionally been addressed through referrals to social workers and community-based organizations (CBOs), but these pathways have had limited success in connecting patients with resources. Given that health inequity is expected to cost the United States nearly USD 300 billion by 2050, new artificial intelligence (AI) technology may aid providers in addressing SDOH. In this commentary, we present our experience with using ChatGPT to obtain SDOH management recommendations for archetypal patients in Philadelphia, PA. ChatGPT identified relevant SDOH resources and provided contact information for local organizations. Future exploration could improve AI prompts and integrate AI into electronic medical records to provide healthcare providers with real-time SDOH recommendations during appointments.

## 1. Introduction

The Quintuple Aim, established in 2021, adds health equity to the previous four pillars of healthcare transformation: patient experience, outcomes, costs, and clinician well-being [1,2]. This model arises from our contemporary understanding of social determinants of health (SDOHs), which account for 70–80% of medical outcomes [2,3,4]. Particularly, the COVID-19 pandemic highlighted prevalent health disparities and questioned the ethicality of the current distributions of resources in our communities [5]. It also demonstrated that equity would produce better health for all members of society [6]. Today, SDOHs are understood to include housing, food, transportation, and more. Each of these impacts the individual and greater society, with implications for the development and prognosis of disease, access to care, and cost.

Health inequity is estimated to cost the U.S. nearly USD 300 billion annually by 2050 [2]. Health systems have invested increasing sums into social programming [7], and there is a burgeoning private industry focused on tackling disparities for patients [4]. Additionally, providers are increasingly expected to add SDOH management to their toolkit. Nonetheless, with demands on physicians’ time rising, overall medical knowledge expanding, and burnout reaching historic levels, it is important to retain realistic expectations for individual physicians. We hypothesized that artificial intelligence (AI) may assist providers with this rising challenge. AI has already shown wide potential for applications in healthcare, including patient education, clinical decision-making support, documentation, and diagnostic assistance within radiology or pathology [8,9,10,11,12]. In this commentary, we present our experience using ChatGPT, the leading generative AI model, to obtain SDOH management recommendations for archetypal patients with health-related social needs reflective of underlying SDOH disparities within our community of Philadelphia, PA.

## 2. ChatGPT Prompt Crafting, Selection, and Accuracy Confirmation

We utilized ChatGPT Version 3.5 to complete a case series evaluation of three archetypal “model patients” who required various SDOH interventions. The models were not based on real patients but were instead designed empirically using commonly reported social concerns among our patient population, which we believe are also applicable to patients in other urban settings. We focused on housing, transportation, and nutritional needs, which are the primary domains defined by Healthy People 2030 [13].

The questions for ChatGPT were developed empirically, framed as typical prompts that would be written by an average physician. The structure of each prompt was as follows: (1) a statement that the question comes from a physician, (2) a brief description of the patient’s chief health complaint, (3) the background of the patient’s living situation and health-related social need, (4) a statement of concern regarding the potential of these needs (that reflect underlying SDOH disparity) to impact the patient’s health, and (5) a request for specific instructions and contact information for resources to address the health-related social need. This prompt structure was created empirically using plain language in consultation with an internal-medicine physician. The patient’s chief health complaint and comorbidities were selected using the most common causes of death, reasons for hospitalization, comorbidities, and long-term diagnoses seen in the county of the study team’s medical center; these were identified using the Pennsylvania Department of Health’s Philadelphia county profile [14]. The three social needs were selected using the Healthy People 2030 domains [13]. Standardized prompt questions were input into ChatGPT as a question-based paragraph (Table 1). Given the stochasticity of sequential generative AI chatbot responses, these prompts were only asked once, and the first response provided by ChatGPT was utilized in the study.

The responses from ChatGPT were generated and reviewed to determine the accuracy of the resources presented. Accuracy was determined by three independent reviewers by confirming that (1) the suggested resources were correctly described and currently operational and (2) the contact information was up to date and correct. The reviewers used the Google search engine first to verify the existence of each suggested resource, and then used that resource’s website, if available, to confirm the program’s mission and contact information. If specific websites did not exist, Google was utilized to further verify this organizational information.

## 3. Representative Responses Obtained from ChatGPT

### 3.1. Analysis of Responses

The responses are presented in Table 1. We found that ChatGPT was able to provide resources relevant to our patients and local community, making recommendations for specific local organizations and even providing contact information. Several resources were provided for all three SDOH domains: housing, transportation, and nutrition. They included multiple programs specific to Philadelphia. Further, they provided actionable advice for the physician, such as detailing the information needed in medical documentation, and often provided contact information or website links for ease of access. Importantly, this was not consistent for all resources, as some were missing phone numbers or website links, and the amount of detail provided for each resource was variable. The order or style of presentation also varied; for instance, websites were largely presented as hyperlinks in the nutrition prompt, while they were given as complete URLs for the other domains.

### 3.2. Verification of Accuracy and Functionality of Resources

Upon performing an online review of the resources provided by ChatGPT, we found 100% of the resources provided to be correctly described, currently operational, and have accurate and up-to-date contact information. There were no defunct, nonexistent, or irrelevant resources provided by the AI chatbot, demonstrating that hallucinations may be less likely in this context, though our small sample size is certainly a limitation.

## 4. Suggestions for Next Steps

### 4.1. Contemporary State of SDOH Intervention

Despite an evolving appreciation for SDOH and an understanding of their costs at a systemic level, there has been little progress in tackling this issue. The current system is likely necessitated by strict time constraints and demands on providers, who are often permitted only 15 min per patient. Traditionally, SDOH-related considerations were handled through referrals to social workers and community-based organizations (CBOs), as providers felt poorly equipped to handle these challenges directly [15]. Unfortunately, CBOs have struggled with funding, reimbursement, and the sharing of patient information, thus hamstringing a potential source of progress backed by local support [4].

There is evidence that considering SDOH needs as a key component of patients’ care plans can improve outcomes and reduce spending [16,17]; this supports increasing agreement that direct physician intervention in social needs is necessary to treat patients holistically [7]. If SDOH truly impact such a large portion of medical outcomes, a culture shift is necessary to ensure that physicians address these factors as strongly as they address biomedical considerations. If physicians are given an efficient and accurate method to identify available resources appropriate to their patients, the burden of referrals and necessary legwork for social workers can be reduced. While our model will not directly tackle the social challenges faced by patients, it can offer an effective first step—one that at least allows for the identification of appropriate resources that the patient can then receive assistance in pursuing.

Under the current screening and referral system, there is a limited success rate in connecting patients with SDOH resources due to inconsistent screening mechanisms [18]. Both screening and intervention often must be performed manually by providers during exceedingly short patient interactions. Studies of the success of SDOH interventions are few. Even at Kaiser Permanente, recognized as a leader in value-based care and health equity, only 23% of referrals resulted in patients’ needs being addressed in the Pacific Northwest [19], while only 10% succeeded in Southern California [20]. Nationally, the Centers for Medicare and Medicaid Services supported a project that screened 1.1 million patients for SDOH needs, but only 14% of those patients received a resolution [21]. Patients who did receive assistance demonstrated significantly reduced emergency department visits and costs [21]. The reasons for this suboptimal success are unclear and may be attributed to poor patient engagement and difficult navigation, along with low perceived importance of these factors by patients and providers alike. These data indicate that health systems must incorporate SDOH management into more routine healthcare practice instead of devolving to third parties.

### 4.2. Potential for AI Solutions

One existing high-quality platform is Findhelp.org, a free search engine that allows patients to quickly locate social service providers in their region. NowPow is a similar program which simplifies referrals and care coordination with community programs, though it is limited to Chicago. We believe that our new AI model offers great potential; with further training, it may provide a similar service to Findhelp.org and NowPow. Future studies may compare the quality of content provided by ChatGPT versus these existing third-party platforms. It is possible that with development, AI can reduce friction via seamless integration into existing physician or patient portals, the automated generation of suggestions and the verification of patient eligibility, and the greater personalization of results; importantly, these features are not currently available. This model can eliminate the need for a middleman and allows physicians to directly intervene without significant burdens on their time. Further, a chatbot platform design is more user-friendly and intuitive, and likely cost-effective as it does not require live staff for database maintenance.

With proper oversight, user training, and further research, medicine can leverage AI to safely augment care quality, facilitate burdensome tasks, and increase face-to-face time with patients [8,9,10,11,12]. These tasks have already been demonstrated [8,9,10,11,12]; in parallel, the existing literature suggests that AI may eventually automatically screen patient charts for SDOH needs, freeing up time during short appointments to discuss this important topic, and tracking patients’ success in navigating resources. This may be beneficial for adherence to all of the Quintuple Aim pillars. Of course, our study does not investigate each these potential uses, but indicates that future study into these realms would be warranted.

Certainly, policymakers must collaborate with providers to refine and expand social services that integrate with health. Of course, several excellent services are already available, yet they are little accessed due to bureaucratic complexity and nebulous qualification criteria. Moreover, resources continuously change with policy and economics. Nationally, there are a variety of services, including financial assistance, food and housing waivers, childcare, utility assistance, and Medicaid. State and local resources vary widely and cannot be adequately discussed in brief publications or provider educational material. Hence, AI can quickly provide physicians with a list of the optimal resources which the physician–patient partnership may then pursue. A future study may include patient-specific qualifiers in the prompt and request that ChatGPT evaluate them for eligibility. Of course, our AI model cannot force the actual connection of a patient with a service, and it is limited to the confines of those resources which already exist.

### 4.3. Cautions for AI Implementation

Nuances in SDOH intervention are rarely, if ever, taught in medical school. It is difficult for a provider, within the short appointment they are allocated, to evaluate a patient for minute qualification criteria and decide the best resource for them. Resources or paperwork that can make a profound difference with a simple physician’s signature are often little known [7]. AI has shown great skill in synthesizing many data points, integrating them, and subsequently providing a range of recommendations. Importantly, providing a list of SDOH-related services or organizations is simply the first step and is not sufficient in effectively addressing current inequities. There remains a need to improve the tracking of successful referrals and their impact on health outcomes to truly measure the impact of these interventions. Structural competency training amongst physicians to help them operationalize these resources is necessary as well, ensuring that physicians work with their patients to implement AI-generated advice while tackling potential obstacles [22].

Of note, there are considerations regarding the limitations of AI. There is extensive literature highlighting the potential for hallucinations, and repeated questioning may yield slightly different responses due to the nature of generative AI [23]. Rarely, this has been shown to contain inaccuracies or misinformation. Further, our study only utilized one style of empiric prompting; further study is necessary to compare results amongst different prompting styles to determine how physicians may craft the most effective questions. Finally, our study did not directly verify the availability and quality of AI-recommended resources with the organizations themselves. Additional studies may include direct communication with the suggested organizations, consultations with local stakeholders, and the assessment of service quality through patient surveys or data analyses of outcomes.

Because AI is based on real-life data, there is potential for the reinforcement of inequity [24]. For instance, the model could learn from online data rooted in structural racism and subsequently make decisions that perpetuate unfairness. A previous study supported this theory that algorithms underlying technological automation may exacerbate existing biases and discrimination; the design of novel tools requires more conscious consideration of systemic racism to prevent this pitfall [24]. The development of new technologies in healthcare must emphasize justice and fairness for all patients. Similarly, historically discriminated-against groups may be less inclined to trust AI, while young and healthy patients gravitate toward this new technology. Nonetheless, studies have shown that patients with underlying social needs have preferred AI interventions to the standard of care [25]. As AI becomes widely available and used in real practice, further refinement can allow it to make physicians’ jobs easier and patients’ experiences more fulfilling.

## 5. Conclusions

Greater adherence to the Quintuple Aim requires commitment by providers to address SDOHs via direct interventions. This presents a unique challenge given the burden of obligation already placed on providers regarding patients’ immediate care needs. The integration of artificial intelligence technology is a potential solution to aid providers in managing SDOH-related challenges beyond the screen-and-refer method that has not sufficiently met patients’ needs. Additional studies are necessary to develop this model, determine its limitations, and reduce the risk of the potential exacerbation of inequity for marginalized populations.

## Figures and Tables

**Table 1:** Representative ChatGPT responses facilitating provider-directed SDOH interventions.

SDOH Category	Question Prompt	Response from ChatGPT v3.5
Housing	I am a physician. My patient in North Philadelphia has severe cardiovascular disease. He has no income and is at risk of becoming homeless because he lost his job. I am concerned this may impact his health. Can you provide specific instructions and contact information that I can use to help my patient obtain stable housing in North Philadelphia?	Of course, I understand your concern for your patient’s health and well-being. Here are specific instructions and contact information you can use to help your patient obtain stable housing in North Philadelphia: **Assessment and Documentation:** Gather comprehensive medical records that highlight the severity of your patient’s cardiovascular disease and the potential health risks associated with homelessness. Document your patient’s loss of income due to job loss and any other relevant social determinants of health impacting their housing stability. **Contact Local Housing Assistance Agencies:** Philadelphia Housing Authority (PHA): PHA manages affordable housing options and rental assistance programs in the city. Website: www.pha.phila.gov Phone: 215-684-4000 **Emergency Shelter and Transitional Housing:** Explore emergency shelters and transitional housing programs that can provide immediate relief while working towards a stable housing solution. Project HOME offers emergency shelters and housing services. Website: www.projecthome.org Phone: 215-232-7272 **Homelessness Prevention Programs:** Look into programs that focus on preventing homelessness by providing financial assistance for rent and utilities. Your patient might qualify for the Emergency Assistance Program through the Department of Human Services (DHS). Website: www.dhs.pa.gov Philadelphia County Assistance Office Phone: 215-560-1900 **211 Helpline:** Encourage your patient to dial 211 for the United Way helpline. They can connect individuals with resources and services, including housing assistance. **Healthcare for the Homeless:** Reach out to organizations specializing in healthcare for homeless individuals. They often have connections to housing resources. Philadelphia Healthcare for the Homeless provides medical and housing assistance. Website: www.projecthome.org/our-work/healthcare Phone: 215-763-6092 **Community Legal Services:** If legal issues are contributing to housing instability, Community Legal Services can provide support. Website: www.clsphila.org Phone: 215-981-3700 **Nonprofit Organizations:** Research local nonprofits like “People’s Emergency Center” that offer housing support to those in need. Website: www.pec-cares.org Phone: 267-777-5820 **Case Management and Social Workers:** Connect your patient with a case manager or social worker who specializes in housing support. They can guide them through the process and advocate for their needs. **Medical Documentation:** Write a letter outlining your patient’s medical condition, the potential health risks of homelessness, and the necessity of stable housing for their well-being. Share this documentation with housing agencies and social service providers. Remember to maintain open communication with your patient, ensuring they actively engage with the resources you provide. The sooner they seek assistance, the better their chances of obtaining stable housing and improving their health outcomes. Keep in mind that contact information and programs may change, so it’s advisable to verify the details before sharing them with your patient.
Transportation	I am a physician. My patient in North Philadelphia has lung cancer and post-traumatic stress disorder. He has limited access to transportation. I am concerned this may impact his health as he cannot easily travel to the clinic or hospital. Can you provide specific instructions and contact information that I can use to help my patient obtain reliable transportation in West Philadelphia?	Certainly, I can provide some general guidance and resources that you can consider for your patient in West Philadelphia. Please note that specific contact information and resources may change over time, so I recommend verifying the information with local organizations or healthcare providers. **Contact Local Hospitals and Clinics:** Reach out to hospitals and clinics in West Philadelphia to inquire about any transportation services they might offer or be aware of for patients with medical conditions. **Non-Emergency Medical Transportation (NEMT) Services:** Look for non-emergency medical transportation services operating in West Philadelphia. These services are designed to assist patients with medical needs. Some well-known NEMT providers include: LogistiCare: (888)-568-0002 MTM, Inc.: (866)-907-1493 **Medicaid Transportation Services:** If your patient is on Medicaid, inquire about transportation benefits. Medicaid often covers transportation to medical appointments for eligible patients. Contact the Pennsylvania Medicaid office for more information. Pennsylvania Medicaid Helpline: 1-800-692-7462 **SEPTA (Southeastern Pennsylvania Transportation Authority):** SEPTA offers public transportation services in the Philadelphia region. It might be a viable option for patients who can use buses, trolleys, and trains. Check SEPTA’s website for routes, schedules, and accessibility information. SEPTA Customer Service: (215)-580-7800 SEPTA Accessible Travel Center: (215)-580-7145 **Ride-Sharing Services:** Consider using ride-sharing services like Uber or Lyft. These services can provide on-demand transportation to medical appointments. **Local Community Centers:** Reach out to local community centers and senior centers. They often have information about transportation resources for individuals with medical needs. **Social Workers and Case Managers:** If your patient has a social worker or case manager, involve them in the process. They might have knowledge of local transportation options. **Local Non-Profit Organizations:** Research non-profit organizations in West Philadelphia that focus on healthcare access, transportation, or support for individuals with medical conditions. **Patient’s Support Network:** Explore whether your patient has family members, friends, or neighbors who might be able to help with transportation. **American Cancer Society:** The American Cancer Society’s “Road to Recovery” program provides transportation to and from cancer-related medical appointments. American Cancer Society: 1-800-227-2345 Remember to ensure your patient’s comfort and safety during transportation, especially considering their medical conditions. Provide them with clear instructions on how to access these services and assistance if needed. Additionally, you can collaborate with your patient’s mental health provider to address their post-traumatic stress disorder and make transportation as stress-free as possible. For the most accurate and up-to-date contact information and resources, I recommend reaching out to local healthcare organizations, social service agencies, or your hospital’s patient advocacy department in West Philadelphia. They can provide you with specific details tailored to your patient’s needs and the current resources available in that area.
Nutrition	I am a physician. My patient in Central Philadelphia has diabetes and osteoarthritis. He has low income and difficulty accessing healthy food. I am concerned this may impact his health. Can you suggest specific organizations that can help my patient obtain access to healthy food in Central Philadelphia?	Certainly, I can provide you with a list of specific organizations and resources in Central Philadelphia that can assist your patient in accessing healthy food despite their low income and health conditions. Please note that availability and services may change over time, so it’s a good idea to verify the information before referring your patient. **SHARE Food Program:** SHARE offers affordable, nutritious groceries through its food package program. Participants can purchase a monthly food package at a reduced cost. Website: SHARE Food Program **Philabundance:** Philabundance is the largest hunger relief organization in the Philadelphia region. They distribute food to individuals and families in need through various programs and partnerships. Website: Philabundance **The Food Trust:** The Food Trust works to increase access to healthy food and promote nutrition education in underserved communities. They operate farmers’ markets, provide nutrition education, and support other food access initiatives. Website: The Food Trust **Metropolitan Area Neighborhood Nutrition Alliance (MANNA):** MANNA delivers nutritious meals to individuals who are battling illness. They offer free meals and nutrition counseling to eligible individuals. Website: MANNA **Greater Philadelphia Coalition Against Hunger:** This organization connects people in need to food resources and provides assistance in applying for SNAP benefits. Website: Coalition Against Hunger **Health Partners Plans:** Health Partners Plans offers a Fresh Food Pharmacy program that provides members with free healthy food to manage chronic conditions like diabetes. Website: Health Partners Plans **Community Clinics and Health Centers:** Many community clinics and health centers in Central Philadelphia collaborate with local food banks and organizations to provide food assistance to patients in need. Check with your patient’s healthcare provider for specific information. **Local Churches and Community Centers:** Churches, community centers, and non-profit organizations often run food distribution programs or provide information about food resources in the area. **Local Farmers’ Markets:** Central Philadelphia has various farmers’ markets that offer fresh produce. Some of them accept SNAP benefits, making it easier for your patient to access healthy food. Remember to advise your patient to contact these organizations directly or visit their websites for more information on how to access their services. You may also want to encourage your patient to work with a registered dietitian to develop a meal plan that aligns with their health conditions and dietary needs.

## Data Availability

The data for this study were obtained from ChatGPT Version 3.5. All ChatGPT data is presented in the table column 3.
